# Tomato root microbiota and *Phytophthora parasitica*-associated disease

**DOI:** 10.1186/s40168-017-0273-7

**Published:** 2017-05-16

**Authors:** Marie Larousse, Corinne Rancurel, Camille Syska, Ferran Palero, Catherine Etienne, Benoît Industri, Xavier Nesme, Marc Bardin, Eric Galiana

**Affiliations:** 1 Université Côte d’Azur, INRA, CNRS, ISA, Sophia Antipolis, France; 2 Centre d’Estudis Avançats de Blanes (CEAB-CSIC), Carrer d’Accés a la Cala Sant Francesc 14, 17300 Blanes, Spain; 3 Université de Lyon, UCBL, CNRS, INRA, Ecologie Microbienne (LEM), 69622 Villeurbanne, France; 4 Plant Pathology, INRA, 84140 Montfavet, France

**Keywords:** Oomycete, Biofilm, Host plant, Metagenomics 16S, *Flavobacteriaceae*, *Pseudomonadaceae*

## Abstract

**Background:**

Interactions between pathogenic oomycetes and microbiota residing on the surface of the host plant root are unknown, despite being critical to inoculum constitution. The nature of these interactions was explored for the polyphagous and telluric species *Phytophthora parasitica*.

**Results:**

Composition of the rhizospheric microbiota of *Solanum lycopersicum* was characterized using deep re-sequencing of 16S rRNA gene to analyze tomato roots either free of or partly covered with *P. parasitica* biofilm. Colonization of the host root surface by the oomycete was associated with a shift in microbial community involving a Bacteroidetes/Proteobacteria transition and *Flavobacteriaceae* as the most abundant family. Identification of members of the *P. parasitica*-associated microbiota interfering with biology and oomycete infection was carried out by screening for bacteria able to (i) grow on a *P. parasitica* extract-based medium (ii), exhibit in vitro probiotic or antibiotic activity towards the oomycete (iii), have an impact on the oomycete infection cycle in a tripartite interaction *S. lycopersicum-P. parasitica-*bacteria. One *Pseudomonas* phylotype was found to exacerbate disease symptoms in tomato plants. The lack of significant gene expression response of *P. parasitica* effectors to *Pseudomonas* suggested that the increase in plant susceptibility was not associated with an increase in virulence. Our results reveal that *Pseudomonas* spp. establishes commensal interactions with the oomycete. Bacteria preferentially colonize the surface of the biofilm rather than the roots, so that they can infect plant cells without any apparent infection of *P. parasitica*.

**Conclusions:**

The presence of the pathogenic oomycete *P. parasitica* in the tomato rhizosphere leads to a shift in the rhizospheric microbiota composition. It contributes to the habitat extension of *Pseudomonas* species mediated through a physical association between the oomycete and the bacteria.

**Electronic supplementary material:**

The online version of this article (doi:10.1186/s40168-017-0273-7) contains supplementary material, which is available to authorized users.

## Background

Microbiota associated either with hosts or/and pathogens regulate the course of infection. For plants, microbiota encompass various functional contexts. This includes stimulation of seed germination and plant growth, promotion of resistance to abiotic stresses, as well as elicitation of plant systemic defense, and antibiosis functions against pathogens [1–5]. For plant pathogens, the incidence of microbe-microbe interactions on virulence is investigated. Studies suggest that fitness results of ability to suppress host defenses and acquire nutrients from host tissues, and also of features that minimize or maximize events associated with undesirable or beneficial co-infections [6, 7]. For example, interactions between microbes may promote pathogenicity by production of bacterial toxins that are essential for fungal virulence or by co-infection events that enhance effector gene expression [6, 8, 9]. Such results contribute to evaluate the updating of our view of pathogenic processes taking into account a broader vision of pathogenesis. Then the question of pathogenicity is not restricted at the single genotype or species level, but is extended at level of microbiota that represent a pathogenic entity [6, 10, 11]. To address these issues, mixed-species biofilms formed by pathogens and resident microbiota in the host vicinity are good models. They constitute an adaptation both for survival, by protecting species from fluctuating conditions, and for molecular dialogs favored by promiscuity between species [12–14].

Pathogenic oomycetes are eukaryotic and filamentous microrganisms infecting various hosts (plants, insects, vertebrates, and other microorganisms) [15, 16]. Plant pathogens of the genera *Phytophthora* and *Pythium*, together with some obligate parasites (downy mildews and white rusts) cause highly destructive diseases on many dicots, thereby having major ecological and economic consequences worldwide [16]. They have evolved the ability to suppress PAMP-triggered immunity (PTI), a key aspect of plant innate immunity which contributes to microbial growth inhibition [17, 18]. Pathogen suppression involves secreted effectors which act in the plant apoplast or are delivered directly into the cytoplasm of host cells, leading to effector-triggered susceptibility [19, 20]. Additionally, oomycetes interact with host resident microbiota before achieving infection. The contribution to pathogenicity of oomycetes/resident microbiota interactions is largely unknown. Recent studies suggest that interactions may act on growth and virulence of the oomycete either at the host–pathogen contact interface or inside host tissues [5, 9]. However, different microorganisms growing in the rhizosphere may exhibit anti-germinative properties against plant-pathogenic oomycetes [21–23], while some inter-specific interactions may promote plant infection [24–26] or may contribute to oomycete propagule dissemination [27]. *Phytophthora parasitica* has been shown to exploit intraspecific zoospore communication to improve adhesion to host cells and to express a set of effector and mucin-like *genes* by means of biofilm formation [28, 29].

A first analysis of the microbiota dynamics in relation to oomycete infections has been carried out in fish [30, 31]. A meta-taxonomic analysis of *Saprolegnia*-associated fish egg diseases indicated a correlation between a low incidence of saprolegniasis on salmon eggs having an immature adaptive immune system and a high richness and abundance of specific commensal Actinobacteria, with species from the *Frondihabitans* genus inhibiting attachment of *Saprolegnia* to eggs [30]. In the case of plant infections, the oomycete-microbiota interactions interfacing with the disease cycle are scarcely documented [6]. In this report, we sought to explore changes in the composition of the rhizosphere microbiota following infection of tomato roots by *P. parasitica*. This issue was addressed through the analysis of interactions occurring within a biofilm formed by the oomycete on the root surface [13, 14]. To test whether there is a subset of the rhizosphere microbiota able to interact with oomycete and affect plant disease onset, a first aim was to characterize composition and specificity of the rhizopheric microbiota having the ability to colonize biofilms. For this purpose, re-sequencing of 16S rRNA gene amplicons was performed to characterize, from phylum to family, content of microbiota resulting from the colonization by soil microorganisms of roots (i) free or (ii) partly covered with a *P. parasitica* biofilm. The second aim was to proceed to culture-dependent functional analyses of the incidence on disease cycle of bacterial species able to colonize the biofilm. Strains were isolated on a *P. parasitica* extract-based medium. They were screened for probiotic or antibiotic activity towards *P. parasitica*, as well as for influence on oomycete pathogenicity in the context of a tripartite host plant-*P. parasitica*-bacteria interaction.

## Methods

### Soil sampling

Sampling was performed on soil supporting tomato growth (*Solanum lycopersicum* cv. Marmande). The experimental site was an E-W-oriented greenhouse located at the INRA Pathologie Végétale research unit in Montfavet (43.9 N, 4.8 E). In this environment, plants were under natural light. Sampling was performed on the 31 May 2014 (10am–2pm) in the following conditions: greenhouse temperature (20, 4–24, 2 °C); air relative humidity (56–73%); soil moisture tension (17–45 cbar). Soil samples were taken from the rhizosphere of 30 plants 6 weeks post-seeding. Plants were randomly selected by groups of three from ten different areas located on two rows, the easternmost and the westernmost within the greenhouse. Samples were then grouped by ten into three biological replicates (*R*_1_, *R*_2_, and *R*_3_) based on the distribution of three samples from each area in distinct replicates. Plants and soil characteristics are described in Additional file 1: Table S1.

### Microbial community constitution

A subsample (10 g) of each biological replicate was mixed with 50 ml of sterile water, and the resulting microbial suspension was decanted for 5 min before recovering supernatants. In order to achieve biofilm formation*,* microbe-free roots of tomato seedlings (2 weeks post germination) were incubated for 3 h at 20 °C with zoospores (10^6^ cells/ml) of the *P. parasitica* strain 149, reported as highly aggressive on tomato [32]. Seedlings were selected under binocular for a biofilm coverage of root surface estimated at 30–50%. Supernatants recovered from microbial suspensions were then placed in a 15-cm plastic Petri dish and were incubated at 20 °C under regular agitation in the presence of tomato roots with or without *P. parasitica* biofilms. Twenty seedlings roots were used to constitute experiment replicates*.* After 3 days, roots were washed 3 times with sterile water. Three replicates of microbial communities physically structured on roots (M1_R1_, M1_R2_, and M1_R3_) or on the root-biofilm complex (M2_R1_, M2_R2_, and M2_R3_) were prepared for metagenomic analysis and screening.

### DNA extraction and high-throughput sequencing

Microbial material for each replicate was obtained via dissociation by mechanical trituration: 20 passes through the opening of a standard Pasteur pipette. DNA was extracted from the homogenate using the FastDNA spin kit for soil (MP Biomedicals, Solon, USA) according to manufacturer’s protocol. Quality of soil DNA was assessed by electrophoresis on 1% agarose, spectrophotometry at 200–300 nm using a NanoDrop 2000 Spectrophotometer (Thermo scientific) and rRNA gene confirmation by PCR using 27F and 1492R primers (Additional file 2: Figure S1). PCR amplification of template DNAs was carried out for the hypervariable V3-V5 region (_~_570 bp length) using the 357F and 926R primers (Additional file 3: Table S2). Conditions for PCR amplification were as previously described [27]. Gel electrophoresis using a 1.5% agarose gel was used to verify amplification. PCR products were excised and purified using the QIAEX II DNA Purification from Agarose Gel kit (QIAGEN) according to manufacturer’s protocol. The quantity of DNA was determined by spectrophotometry (Nanodrop 2000 spectrophotometer). Amplicon libraries were constructed for each replicate using the InViewTM Microbiome Profiling 2.0 service and paired-end sequenced with Illumina MiSeq sequencing at GATC Biotech (Konstanz, Germany).

### Treatment and phylogenetic classification

Sequence analysis was performed using the Ribosomal Database Project tools, following RDP Release 11.4 recommendations [33]. Sequences generated from the 357F primer were subjected to quality filtering with FastQC for average percentage of A, G, C, and T across the read length, average GC content, location and frequency of N positions, sequence length distribution, duplicate sequences, and overrepresented k-mer sequences. Utilizing quality scores, sequence reads were cleaned by PRINSEQ and RDP. Initial processing used criteria: removal of 5 and 74 nucleotide residues at the 3′ and 5′ ends, respectively; deletion of reads with N residues or with an average quality score ≥32. Chimeric sequences (*n* = 64108) were also deleted after identification with UCHIIME [34] via USEARCH [35] and against the RDP Gold v9 database. After processing, 644019 sequence reads (including the V3 region) were selected for further analyses. Alignment was performed using the HMMER3 model [36]. The RDP’s mcClust algorithm [37] and the naïve Bayesian classifier [38] were used to define 38730 operational taxonomic units (OTUs) at 98% sequence identity. A multivariate data analysis of OTUs was performed using Phyloseq. Principal component analysis (PCA) and significant features were identified for all treatments using Phyloseq. The R Vegan package [39] was used for community dissimilarity calculations (alpha diversity indices combining species richness and abundance into a single value) with for each replicate a OTU number normalized relative to the lowest number of OTUs generated from M2_R1_. The Shannon–Weaver index was ranged from 0.74 to 0.88 for M1_R1_, M1_R2_, and M_1R3_ and from 0.77 to 0.87 for M2_R1,_ M2_R2_, and M2_R3_. The Simpson index was ranged from 0.41 to 0.52 and from 0.51 to 0.52 for M1 and M2 replicates, respectively (Additional file 4: Figure S2).

### Root-biofilm complex (M2) screening

Bacterial isolates were generated as described by Galiana et al*.* [27]. Mixed-species biofilms were recovered from M2, were rinsed three times in water, and were gently dissociated by mechanical trituration (as described above). Cell suspensions obtained were spread on agar plates containing a *Phytophthora* extract as sole nutrient source [*Phytophthora* crude extract 10 g/l; NaCl 10 g/l; agar 1.5% (P/V)] and were incubated at 25 °C. *Phytophthora* crude extract was prepared from a 2-week mycelium of *P. parasitica* strain 149 (INRA, Sophia Antipolis, France). Colonies appeared within 3 days. After subculturing in the same conditions, bacteria were transferred to LB medium for mass culture and further analyses.

### Characterization of genetic diversity by 16S rRNA gene sequencing

Bacteria were grown in LB medium over night at 28 °C. Genomic DNA was extracted from cell cultures using the UltraClean®Microbial DNA Isolation kit (MO BIO). Extracted DNA was amplified with 27F and 1492R primers. Amplification was performed under the following conditions: initial denaturation step at 94 °C for 5 min; 40 cycles of denaturation at 94 °C for 40 s, annealing at 55 °C for 40 s, and extension at 72 °C for 1.5 min; and a final extension at 72 °C for 7 min. PCR products were separated on a 1% agarose gel, were stained with ethidium bromide, and were visualized on a transilluminator. Sequencing was performed in both directions with primers 27F and 1492R, and consensus sequences were obtained using CAP3 [40]. Identification was performed at the genus level by blast against the “procaryota_SSU-rDNA-16S_stringent 277957” leBIBI-QBPP database (https://umr5558-bibiserv.univ-lyon1.fr/lebibi/lebibi.cgi). 16S rRNA gene sequences closest to the isolates (98% sequence homology) were recovered for phylogenetic analysis. Two groups were formed and analyzed separately per higher-level taxonomy. Sequence alignment was performed with MUSCLE [41], and the phylogenetic tree was constructed using the GTR model in PhyML [42] as implemented in the software Seaview4. A bootstrap confidence analysis was performed with 1000 replicates.

### Generation of green fluorescent protein-labeled bacterial strains

Bacteria were grown in LB medium and washed with ice-cold 10% glycerol. The pFK78 plasmid [43] was transferred into competent cells using the MicroPulser™ electroporation apparatus (Bio-Rad) with recommended protocols for bacteria. Transformed bacteria were selected on LB agar plates supplemented with gentamycin (10 μg ml^−1^) and identified using a LEICA MZFLIII binocular and AxioCamHR camera equipped with AxioVision 4_7 software (Zeiss, Germany). GFP was excited at 440–520 nm light and emission was detected through a 520–600 GFP filter.

### Biofilm colonization assay

To generate biofilm, roots of tomato seedlings were inoculated for 3 h with 10 ml of *P. parasitica* zoospore suspension (strain 149, 500 cells μl^−1^) and were washed three times with sterile water [28]. The biofilm-root complex was inoculated with GFP-expressing bacteria (*E. coli* and I-1G6) by adding 10 ml of a cell suspension in water (OD = 0.2) and was incubated for 3 h at 20 °C. Analysis of biofilm-root colonization was performed at 20 °C and was visualized at different time points (3, 6, 18, 24, 48, 72, and 96 h) by fluorescence microscopy. For quantitative image acquisition, 8 bit images (512 × 512 pixels) were acquired on a ZEISS LSM 880 laser scanning confocal microscope (λex = 488 nm). In order to measure root and biofilm colonization, a mean fluorescent intensity signal was determined for four 1000 μm^2^ areas on each sample (2-biofilm and 2-root material), from 10 serial confocal sections (5 μm) using the ZEN 2012 lite software (Zeiss, Germany).

### Tripartite inoculation and protection assay

Roots were inoculated for 3 h with 10 ml of *P. parasitica* zoospore suspension (strain 149, 10 cells μl^−1^), were washed three times with sterile water, and were inoculated with bacterial isolates suspended in sterile water (OD = 0.2). Plants were grown at 24 °C under growth chamber conditions; 16 h photoperiod at a light intensity of 100 mEm^−2^ s^−1^. Disease incidence was measured at different time points as the percentage of plantlets exhibiting symptoms (yellowed leaves, root rot, and plant stunting). Experiment was performed using two replicates of five plants each.

### Real-time qPCR analyses

Gene expression was quantified by real-time RT-qPCR using the fluorescent intercalating dye SYBR-Green in an AriaMx Realtime PCR System (Agilent Technologies, Santa Clara, USA). Total RNA from the bacteria-biofilm-root complex was isolated with TRIzol reagent (Invitrogen GmbH, Karlsruhe, Germany). mRNA was treated with Ambion® rDNase I (Thermo Fisher Scientific, Waltham, USA), and cDNA was synthesized from 1 μg RNA, by iScript cDNA Synthesis (Bio-Rad, Hercules, USA). The cDNA was used as a template in real-time PCR with gene-specific primers (Additional file 3: Table S2) and the qPCRTM Mastermix Plus for SybrTM Green I (Eurogentec, Belgium), following the manufacturer’s instructions. PCR amplification and statistical analyses was carried out as previously described [29].

## Results

### Structure of *P. parasitica*-associated microbiota

**Fig. 1:**
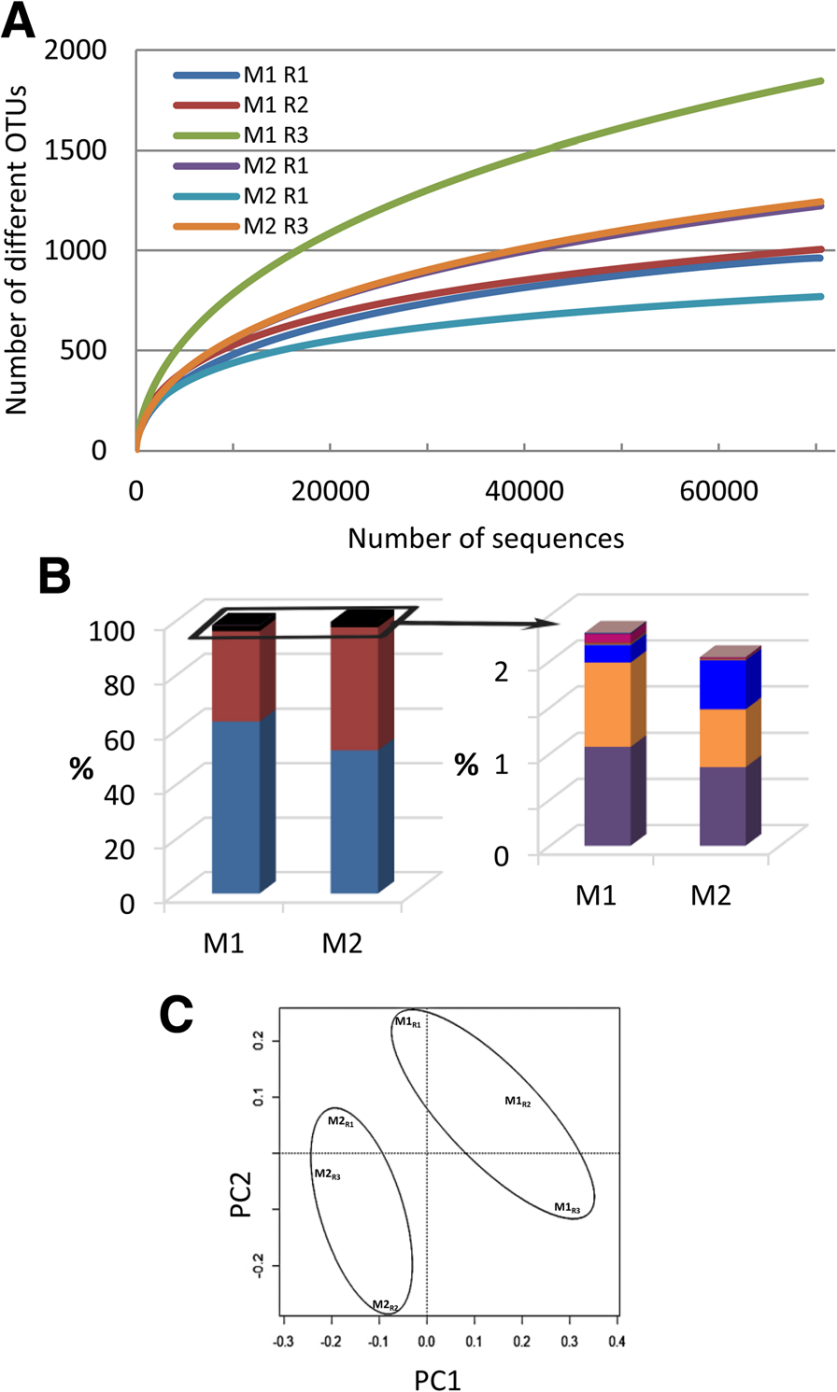
**a** Rarefaction curves of observed OTUs richness for each of the three M1 and M2 replicates and using an OTU threshold of ≥97% identity. **b** Percentage of high-confidence OTUs grouped by phylum: Proteobacteria (*blue*) and Bacteroidetes (*red*) (*left* panel); Latescibacteria (*purple*), Actinobacteria (*orange*), Firmicutes (*blue*), Verrucomicrobia (*dark pink*) (*right* panel). **c** PCA ordination based on Hellinger distances

Metagenomic analyses allowed characterization of rhizosphere-associated microbiota colonizing roots of tomato seedlings without (M1) or previously coated with *P. parasitica* (M2). Rarefaction curves for six replicates (M1_R1–3_ and M2_R1–3_) using a 3% dissimilarity cut-off were non-asymptotic in spite of a good coverage (Fig. 1a). Thirty-eight 730 bacterial operational taxonomic units (OTUs) were detected in the 6 samples. The overall microbial diversity of all samples was relatively high, but no reliable differences were identified between rhizospheric microbiota of M1 and M2 (Additional file 4: Figure S2). The most abundant groups were adequately covered in all samples, with over 97% of sequences assigned to Proteobacteria or Bacteroidetes along with a limited number of OTUs assigned to additional phyla, including Firmicutes and Actinobacteria (Fig. 1b). As a matter of fact, all these phyla are known to characterize a number of rhizosphere microbiota [2, 44], indicating that the microbiota we studied have the same overall content as that of other rhizosphere microbiota. The relative contents of all samples were highly correlated (data not shown); nevertheless, PCA ordination as well as hierarchical clustering revealed that M1 and M2 samples separated (Fig. 1c). A total of 322 OTUs exhibited significant differences (*p* < 0.05) in relative abundance between M1 and M2 (Additional file 5: Figure S3), with 49 OTUs defined by an average of at least 10 sequences in M1 or M2 (data not shown).

**Fig. 2:**
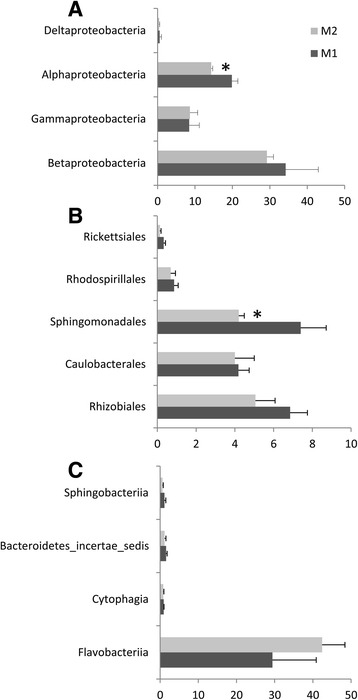
Relative abundance histograms (percentage ± SD) of Proteobacteria (**a**), Alphaproteobacteria orders (**b**), and Bacteroidetes classes (**c**) present in M1 or M2. Differences between M1 and M2 were significant for Alphaproteobacteria (*p* = 0.03) and Sphingomonadales (*p* = 0.05) in a Student’s *t* test (*n* = 3)

**Fig. 3:**
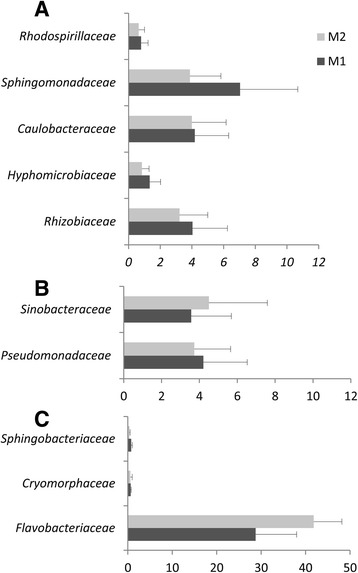
Relative abundance histograms (percentage + SD) of the ten most abundant families listed for M1 and M2 (threshold >0.5%) in the presence (*light gray*) and in the absence (*dark gray*) of *P. parasitica* biofilm. **a** Alphaproteobacteria. **b** Gammaproteobacteria. **c** Bacteroidetes

Our results suggest that the presence of oomycete on root surfaces led to an enrichment of sequences assigned belonging to Bacteroidetes (33% ± 10.8, 45% ± 5.2 for M1 and M2, respectively), and a reduction in the proportion of sequences assigned to the phylum Proteobacteria (63% ± 10.1, 52% ±3.9 for M1 and M2, respectively). Furthermore, our results indicate an effect of oomycetes on rhizosphere microbiota capable of colonizing tomato root. The distribution of sequences assigned to the four classes of Proteobacteria showed a significant reduction (*p* = 0.03) in Alphaproteobacteria abundance (Fig. 2a); a reduction largely supported by a significant lower number of sequences assigned to the order Sphingomonadales (*p* = 0.05) (Fig. 2b). A reduction in the relative abundance of Alphaproteobacteria families was also found to be associated with the presence of oomycete for *Sphingomonadaceae* (*p* = 0.04) in the order Sphingomonadales, and for *Hyphomicrobiaceae* (*p* = 0.02) and *Bradyrhizobiaceae* (*p* = 0.03) in the order Rhizobiales (Fig. 3). Within Bacteroidetes, most OTUs found in both M1 and M2 were related to Flavobacteriia (Fig. 2c) with *Flavobacteriaceae* as the most abundant family (Fig. 3c). *Flavobacteriaceae* supports thus the higher relative abundance of Bacteroidetes found associated with the presence of oomycete (M2 vs. M1, *p* = 0.07). The overall taxonomic assignment of annotated rRNA gene sequences at the family level and relative percentage in M1 and M2 are shown in Additional file 6: Table S3. Ten families explain 55.3 and 63.5% of the microbial diversity both in M1 and M2, respectively (Fig. 3). The range of 16S gene copy number in the genome is given for each of these families in Additional file 7: Table S4 wherever data was available [45].

### Partnerships between *P. parasitica* and bacteria M2

**Fig. 4:**
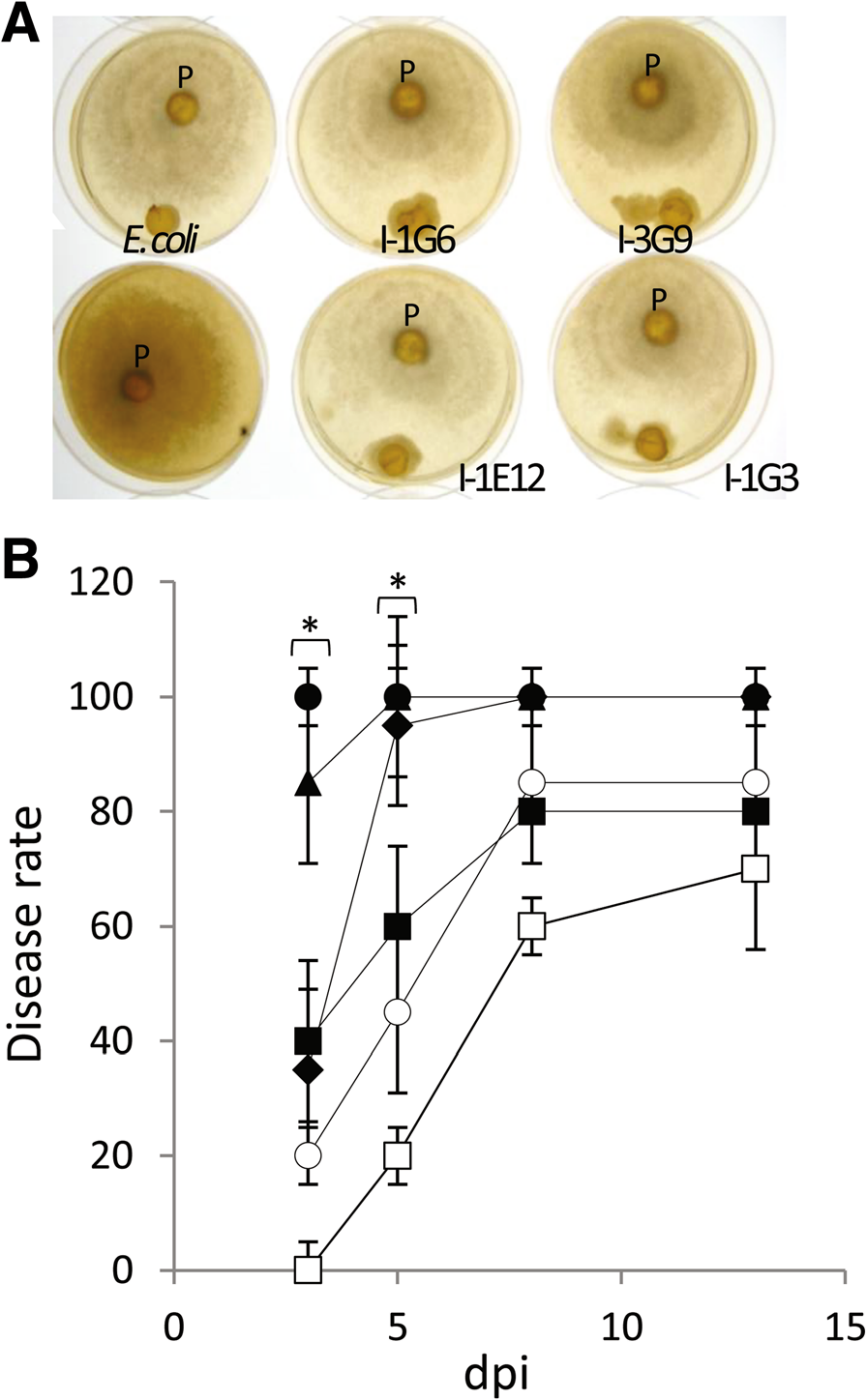
Bacterial isolate analyses. **a** Plate confrontation assay of the *E. coli* strain and the M2-isolates (I-1G6, I-3G9, I-1E12, and I-1G3) exhibiting antimicrobial activity against *P. parasitica* (*P*) performed on V8 extract agar medium. **b** Histogram of the disease rate measured at different days post zoospore inoculation (○) or during a tripartite interaction with *E. coli* (□),I-3G9 (●), I-1E12 (▲), I-1G6 (♦), or I-1G3 (■). Disease symptoms of individual plants were monitored at 3, 5, 8, and 13 days (dpi). I-3G9 as well as I-1E12 differed from *E.coli* and mock inoculation in disease index measured at 3 and 5 dpi (*n* = 5, Student’s *t* test, 0.004 < *p* < 0.035). Values are the means ± SD of two replicates

M2 replicates were screened for bacterial species associated with *P. parasitica* and regulated plant infection*.* After dissection and dilaceration of mixed-biofilms, a collection of 1200 isolates was recovered from M2 on a *P. parasitica* crude extract-based medium. A total of 300 isolates were tested for activity towards the in vitro and in planta growth of the oomycete. Eleven percent of the isolates inhibited (9.8%) or promoted (1.2%) radial growth of three distinct *P. parasitica* strains in a confrontation assay on nutrient agar plates (Fig. 4a). A similar tendency was observed for the growth effect in zoospore germination assay and in the presence of isolate-conditioned V8 medium for each of these isolates (data not shown). Our results suggest in vitro isolates that potentially secrete compounds which interfere either negatively or positively with *P. parasitica* growth. Sequencing of 16S rRNA genes and searches of the procaryota_SSU-rDNA-16S_stringent 277957 database of the leBIBI-QBPP [46] established that isolates belonged to three phylotypes of *Pseudomonas* spp. and one phylotype of *Enterobacter* spp. (Additional file 8: Figure S4). In the M2 treatment, the mean proportion of OTUs affiliated to *Pseudomonadaceae* or *Enterobacteriaceae* was 3.7 and 0.009%, respectively.

Twenty isolates were tested using a co-inoculation assay system for detecting their effect on the ability of a virulent *P. parasitica* strain to infect tomato roots. Each individual isolate was applied directly to the roots, none was found to cause symptoms on leaves or roots of plantlets for periods of up to 14 days (see Additional file 9: Figure S5 for *Pseudomonas* isolates). No correlation with the in vitro effect of isolates on the oomycete growth were observed. Indeed, for all isolates similar root application at 2 h post-zoospore inoculation did not lead to curative protection of plants (data not shown). For four isolates belonging to the *Pseudomonas* phylotype II and III worsening of symptoms was noted. The rate at which the symptoms appeared and progressed was faster than the appearance of symptoms in control plants inoculated with zoospores or co-inoculated with zoospores and cells of *E. coli* lab strain. The disease rate was significantly higher for two isolates at 3 and 5 days post-inoculation (Fig. 4b).

### *Pseudomonas* spp. preferentially colonizes *P. parasitica* biofilm and infects plant cells

**Fig. 5:**
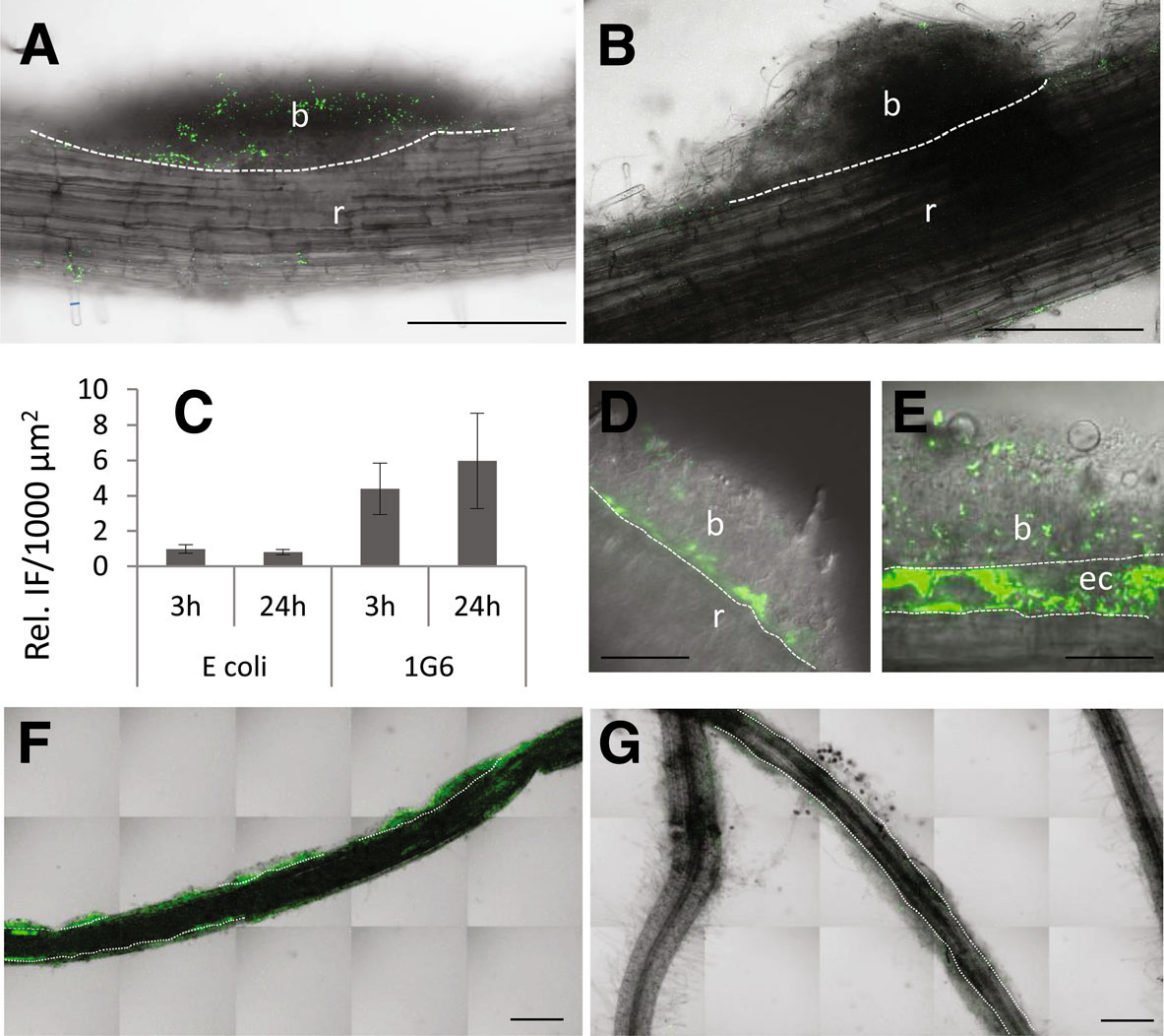
Location of I-1G6-GFP. Micrographs illustrating the preferential location of 1G6-GFP cells on biofilm (*b*) compared to root (*r*) at 3 hpi (**a**), 48 hpi (**d**, **e**), and 8 dpi (**f**). The colonization observed for I-1G6-GFP at 3 hpi (**a**) 8 dpi (**f**) can be compared to the lesser one observed for *E. coli*-GFP (**b**) and (**g**), respectively. **c** Histogram of the relative fluorescence intensity at 3 and 24 hpi for I-1G6-GFP and *E.coli*-GFP, measured as the ratio of the mean values at the surface of the biofilms and of roots. *Bars*: 200 μm in **a**, **b**, **f**, and **g**; 50 μm in **d** and **e**. The *dotted lines* delineate the interface between biofilm and root. In (**e**) *dotted lines* delineate an epidermal cell infected by I-1G6-GFP-and located beneath a biofilm

In order to further analyze the root-*P. parasitica*-*Pseudomonas* spp. association, a GFP expressing strain I-1G6-GFP was generated. A kinetic study was then carried out by immersing tomato roots covered with biofilm in a bacterial suspension of I-1G6-GFP or an *E. coli* expressing GFP. Samples were observed under a confocal microscope at different times (3, 24, and 48 h post-inoculation or hpi). Fig. 5a and Additional file 10: Figure S6, show micrographs with the presence of bacteria at 3 hpi. At this early stage the I-1G6-GFP bacterial cells were poorly distributed along root surface and were mainly located on biofilm-forming hemispherical or sleeve-shaped biofilms. Quantification of fluorescence intensities showed that the colonization of biofilms was preferential when compared to root surfaces for I-1G6-GFP (Fig. 5c). At 24 and 48 hpi, similar observations were made with an increase of colonization on biofilm surfaces by I-1G6-GFP, suggesting a phase of cell division at the surface of the biofilm. For the *E. coli-*GFP strain, the colonization of biofilms surface was sparser and unspecific compared to root surface (Fig. 5b, c). At 2 dpi, I-1G6-GFP bacteria may have accumulated at the interface between the root surface and the structure of the biofilm (Fig. 5d), and infection of plant cells adjacent to the biofilm was also observed (Fig. 5e). At 8 dpi, preferential colonization of biofilm was well marked (Fig. 5f, g), extensive intercellular growth of I-1G6-GFP was observed in the root cortex, mainly along the longitudinal axis of the root (Additional file 11: Figure S7). At different time points, conversely to plant cell, no intracellular infection event of *P. parasitica* could be observed.

### *Pseudomonas* spp. does not induce *P. parasitica* effector gene regulation

**Fig. 6:**
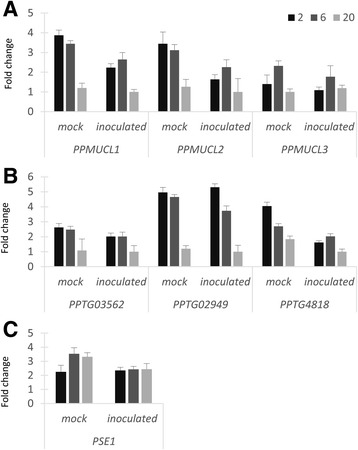
*P. parasitica* gene expression patterns in response to I-1G6-GFP. Roots of 2-week-old plants covered with a *P. parasitica* biofilm after 3 h of incubation with zoospores. They were then incubated in water or in the presence of I-1G6-GFP. mRNAs relative abundance were measured by RT-qPCR at 2, 6, and 20 h post-bacterial inoculation and for *PPMUCL1*, *PPMUCL2*, *PPMUCL3* (**a**), *PPTG03562*, *PPTG02949*, *PPTG4818* (**b**) and *PSE1* (**c**). A Student’s *t* test indicated no significant differences between mock and inoculated situations (0.06 < *p* < 0.92, *n* = 3) except at 2 dpi for *PPMUCL1*, *PPMUCL2*, and *PPTG4818* (*p* = 0.01, 0.02, and 0.04, respectively)

Previous investigations on the *P. parasitica* biofilm transcriptome led to the identification of upregulated transcripts encoding (i) mucin-like proteins of the *PPMUCL* family [29], (ii) pectate lyases (*PPTG03562/02949/4818*) identified as involved in the degradation of pectin, components of the plant cell wall [47], and the RxLR effector *PSE1* accumulating in penetrating appressoria [48]. To characterize the effect of *Pseudomonas spp.* gene expression at early stages of infection, a kinetic study was performed by quantitative RT-PCR during colonization of the biofilm-root complex by I-1G6. Fig. 6 shows the mRNA changes for the three classes of genes at the different time points. The presence of I-1G6 strain does not appear to affect the expression levels of the genes despite a slight reduction in expression levels 2–20 h after colonization. Our results suggest that, at early stages of tripartite interaction, the abundance of assessed transcripts were not upregulated in response to the colonization of the biofilm by *Pseudomonas spp.*

## Discussion

### The Bacteroidetes/Proteobacteria shift

Here, we report the change in the composition of the rhizosphere microbiota of *Solanum lycopersicum* at root surface in association with *P. parasitica* biofilm formation, at phylum (Fig. 1b), class, order (Fig. 2), and family (Fig. 3) level assignments. Among ten families characterized as the most abundant in M1 and M2 (Fig. 3), *Rhodospirillaceae*, *Pseudomonadaceae*, and *Flavobacteriaceae* exhibit a high range of 16S gene copy number in their genomes, with an average of 4.5, 4.8, and 3.9, respectively (Additional file 7: Table S4). This indicates that the relative abundance values in M1 and M2 may not be an accurate representation for these families. Nevertheless, *Flavobacteriaceae* remains the most abundant family, with a representation eight to ten times higher compared to the other nine main families identified.

Our results suggest a *P. parasitica*-associated shift involving a Bacteroidetes/Proteobacteria transition in microbiota composition at the root surface. The Bacteroidetes dominate colonization of tomato roots inoculated with *P. parasitica*. The infection of a host plant by *P. parasitica* relies on the secretion of plant cell wall-degrading enzymes that leads to successful penetration of the host and to subsequent acquisition of nutrients [47]. The ability of the oomycetes to efficiently depolymerize polysaccharides could contribute to the observed enrichment of Bacteroidetes within *P. parasitica*-associated microbiota. Research indicates that the abundance of Bacteroidetes in soils is positively correlated to carbon mineralization rates [49], influenced by oomycetes through pectin digestion [50]. Localized degradation of plant cell wall at root sites of biofilm formation should constitute a favorable niche for soil *Flavobacteriaceae*, the predominant family found in M2. Furthermore, within *Flavobacteriaceae* family, genomes exhibit a high abundance and diversity of genes involved in metabolism of carbohydrates such as xylose, arabinose, and pectin [51]. The increased expression of three genes encoding *P. parasitica* pectate lyases (Fig. 6b), included in the top 20 most expressed genes in biofilm (present study and [52]), is consistent with a causal relationship between *P. parasitica* pectin digestion ability and Bacteroidetes enrichment.

From a pathological perspective, the question of if/how Bacteroidetes/Proteobacteria community shift interferes with the infection cycle of oomycetes remains open. Our research functionally assessed the roles of *P. parasitica*-associated bacteria and tested hypotheses generated via culture-independent profiling. Screening of root-biofilm complex M2 led us to characterize members of *Pseudomonadaceae*, one of the ten most abundant families of the microbiota (OTU affiliation rate of 3.7%), and isolates of *Enterobacteriaceae*; a family representing a low part of M2 microbial diversity (OTU affiliation rate of 0.009%, data not shown). While no Bacteroidetes strains were characterized from M2 with the employed strategy. Further investigations are required to design effective screening for isolation of Bacteroidetes strains from the *P. parasitica*-associated microbiota [53].

### Effects of *Pseudomonas* spp*.-P. parasitica* association on pathogenicity

Associations with bacteria can have a considerable influence on the growth, physiology, and pathogenicity of filamentous pathogens [8]. Previous studies reported that bacteria growing in the rhizosphere may compete with oomycetes for nutrients or may exhibit properties that negatively affect growth/survival. For example, competition for plant-derived unsaturated long-chain fatty acids has been reported between *Enterobacter cloacae* and the seed-rotting oomycete, *Pythium ultimum* [22]. Here, the interference of *Pseudomonas* spp. and *Enterobacter* spp. on the biology of oomycete was assessed both in vitro and in planta. Interactions via antibiosis and probiosis were clearly observed in vitro for members of two bacterial genera and involving secreted compounds. Our results indicate that bacteria-oomycete interactions, in the rhizosphere, may have detrimental or beneficial impact on the growth of the stramenopile. However, in the context of tripartite interaction *S. lycopersicum-P. parasitica*–bacteria strain, the activities characterized in vitro were not expressed or had no influence on the ability of the oomycete to colonize a host plant.

We identified one *Pseudomonas* phylotype (exhibiting antibiosis activity), which significantly increased symptom severity on treated plantlets. When inoculated alone (without *P. parasitica*), the phylotype never provoked visible symptoms (Additional file 9: Figure S5D). These results suggest either an enhancement of the *P. parasitica* pathogenicity by bacteria or a potentiation of the infectiveness/pathogenicity of *Pseudomonas* spp. by the oomycete. Further studies are required to discriminate among these alternative hypotheses. Nevertheless, our results suggest that oomycete infection may facilitate opportunistic infection by *Pseudomonas* spp. The first assumption has been evaluated at the transcriptomic level through the analysis of gene expression of targeted genes recruited for structural functions [29] or by brute force [54] and stealth [48] modes of infection [54]. No influence of the bacteria-oomycete interaction was observed on the mRNA abundance of genes during the oomycete infection process. On the other hand, the second assumption is supported by the location of bacteria at the *P. parasitica* infection site which is favored by the preferential adhesion on oomycete material. It is also supported by the occurrence of subsequent *Pseudomonas* infections of adjacent plant cells. *Pseudomonas* opportunistic pathogens may have acquired the ability to adhere to the oomycete to maximize access to plant nutrients. At wound sites [43, 55], the oomycete material would constitute a boarding gate for bacteria of this family to gain access to nutrients.

## Conclusions

Significant changes in microbiota composition during the oomycete plant infection were identified. Establishment of host-oomycete interaction is characterized by a higher relative abundance of taxa within Bacteroidetes and a lower relative abundance of Proteobacteria. The present study further illustrates an aspect of cooperation between an oomycete and opportunistic bacteria by demonstrating that oomycete infection extends the habitat availability for *Pseudomonas* spp. to host-plant tissues. Future mechanistic insights into bacterial adhesion on oomycete surfaces and possible optimization of resource allocation due to infection will be obtained through microbial genetics and functional analyses.

## Additional files

Additional file 1: Table S1. Plant characteristics and soil properties at each sampling location. (PDF 348 kb)

Additional file 2: Figure S1. PCR analysis of DNA extracted from replicates M1R_1–3_ and M2R_1–3_. PCR amplificons corresponding to full-length *16S* rRNA gene were generated using 27F and 1492R primers and analyzed by 1% agarose gel electrophoresis. Lane MW corresponds to molecular-weight size markers (MassRuler DNA Ladder Mix, Thermo Scientific). (PDF 5664 kb)

Additional file 3: Table S2. Nucleotide sequences (5-3′) of the primers used in this study. (PDF 451 kb)

Additional file 4: Figure S2. Graphic summary of alpha-diversity indices**.** Comparison of Chao1, Shannon, Fisher, and Simpson indices calculated for M1 and M2 replicates. (PDF 13600 kb)

Additional file 5: Figure S3. Dendrogram showing hierarchical clustering of the six biological replicates using the unweighted pair-group average algorithm and the Bray–Curtis similarity index (*n* = 1000). The dendogram is drawn based on the relative abundances of OTUs showing significant difference between M1 and M2 (*p* < 0.05). (PDF 695 kb)

Additional file 6: Table S3. Rhizosphere bacterial families identified in M1 or M2, and responded to the presence of *P. parasitica* biofilm. (PDF 655 kb)

Additional file 7: Table S4. 16S gene count statistics (20 March 2017). (PDF 445 kb)

Additional file 8: Figure S4. Phylogenetic trees showing the relationship between the *Pseudomonadaceae* (**A**) and *Enterobacteriaceae* (**B**) isolates from M2 and related *Pseudomonadaceae* or *Enterobacteriaceae* species, respectively. Trees were constructed using PhyML with default settings and 1000 bootstrap replicates. The position of rhizospheric isolates are indicated by vertical bars. (PDF 7876 kb)

Additional file 9: Figure S5. Two-week-old plants were first root-inoculated (**A**, **C**) or not inoculated (**B**, **D**) with a 10 ml suspension of zoospores (2 cells/μl) from the *P. parasitica* strain 149. Two replicates of five plants were subsequently inoculated with the indicated bacterial isolates and photographed 8 days post-inoculation (**C**, **D**). As shown in (**C**), when inoculated alone the tested isolates (I-3G9, I-1G6, I-1G3) did not cause visible disease symptoms on plants. As illustrated in (**C**), the co-inoculation of rhizospheric isolates with *P. parasitica* led to aggravation of symptoms when compared to the co-inoculation of *E. coli* cells with *P. parasitica* zoospores. (PDF 31934 kb)

Additional file 10: Figure S6. Three representative optical section Z-series illustrating the preferential location of 1G6-GFP cells on different *P. parasitica* biofilms (b) formed on the surface of roots (r) (3 hpi). Fluorescence intensities were measured on ten consecutive serial sections (5 μm), (i) at the *left* and *right* parts of the biofilm (1000 μm^2^), and (ii) at the root surface not covered by *P. parasitica* and located on the *left* and *right* sides of the biofilm (1000 μm^2^). *Bars*: 100 μm in **A** and **C**; 200 μm in **B**. (PDF 29611 kb)

Additional file 11: Figure S7. Location of I-1G6-GFP in the root cortex at 8 dpi. (**A**, **B**, **C**) Roots nude (**A**) or recovered with *P. parasitica* biofilm (**B**, **C**) inoculated with *E. coli*-GFP cells. (**D**, **E**, **F**) Roots nude (**D**) or roots covered with *P. parasitica* biofilm (**E**, **F**) and inoculated with I-1G6-GFP. (**G**) Enlargement of the two inlets indicated in F, showing root cortex colonization along the longitudinal axis of the xylem. *Bars*: 100 μm. (PDF 24571 kb)
