# Supplementary material for: Tomato root microbiota and Phytophthora parasitica-associated disease
Source: Microbiome. 2017 May 16;5:56. doi: 10.1186/s40168-017-0273-7 (PMC5434524; doi:10.1186/s40168-017-0273-7)
Supplement: Supplementary file 1 — Plant characteristics and soil properties at each sampling location. (PDF 348 kb) [file 40168_2017_273_MOESM1_ESM.pdf]

Table S1 Plant characteristics and soil properties at each sampling location

Replicates	Sample number	Plant size (cm)	Number of fruits	Soil temperature (°C)	Soil pH
**R1**	1	85	8	17,5	7,81
	2	70	15	17,9	8,09
	3	88	13	18,1	8,56
	4	82	12	18	8,45
	5	82	8	18	8,32
	6	83	14	18,8	8,66
	7	75	17	21	8,51
	8	73	15	22	8,54
	9	80	17	21	8,66
	10	75	16	20	8,37
**Mean R1**		**79,3**	**13,5**	**19,2**	**8,4**
**SD R1**		**5,8**	**3,3**	**1,6**	**0,3**
**R2**	11	85	16	17,2	8,33
	12	75	10	17,9	8,49
	13	87	11	18,1	8,18
	14	87	19	17,8	8,36
	15	85	14	18,4	8,45
	16	83	18	19	8,46
	17	75	13	22,4	8,39
	18	80	14	21,2	8,28
	19	81	20	21	8,42
	20	78	21	19,6	8,71
**Mean R2**		**81,6**	**15,6**	**19,3**	**8,4**
**SD R2**		**4,6**	**3,8**	**1,7**	**0,1**
**R3**	21	84	18	17,6	8,2
	22	70	14	17,5	8,5
	23	77	16	17,9	8,08
	24	80	13	18,1	8,55
	25	85	15	18,3	8,42
	26	73	12	21,1	8,39
	27	80	16	22,9	8,07
	28	70	12	21,6	8,37
	29	83	17	21	8,72
	30	81	16	20,1	8,7
**Mean R3**		**78,3**	**14,9**	**19,6**	**8,4**
**SD R3**		**5,6**	**2,1**	**2,0**	**0,2**
