# Supplementary figures and images for: Tomato root microbiota and Phytophthora parasitica-associated disease
Source: Microbiome. 2017 May 16;5:56. doi: 10.1186/s40168-017-0273-7 (PMC5434524; doi:10.1186/s40168-017-0273-7)

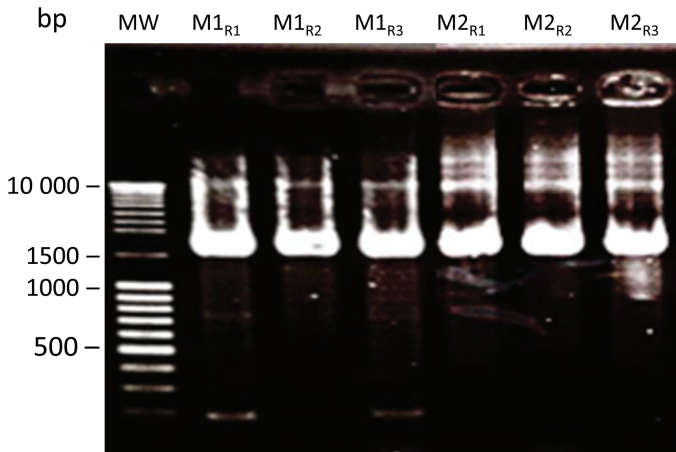

Supplement: Supplementary file 2 — PCR analysis of DNA extracted from replicates M1R1–3 and M2R1–3. PCR amplificons corresponding to full-length 16S rRNA gene were generated using 27F and 1492R primers and analyzed by 1% agarose gel electrophoresis. Lane MW corresponds to molecular-weight size markers (MassRuler DNA Ladder Mix, Thermo Scientific). (PDF 5664 kb) [file 40168_2017_273_MOESM2_ESM.pdf]

similarity

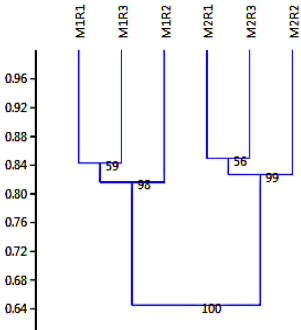

Supplement: Supplementary file 5 — Dendrogram showing hierarchical clustering of the six biological replicates using the unweighted pair-group average algorithm and the Bray–Curtis similarity index (n = 1000). The dendogram is drawn based on the relative abundances of OTUs showing significant difference between M1 and M2 (p < 0.05). (PDF 695 kb) [file 40168_2017_273_MOESM5_ESM.pdf]

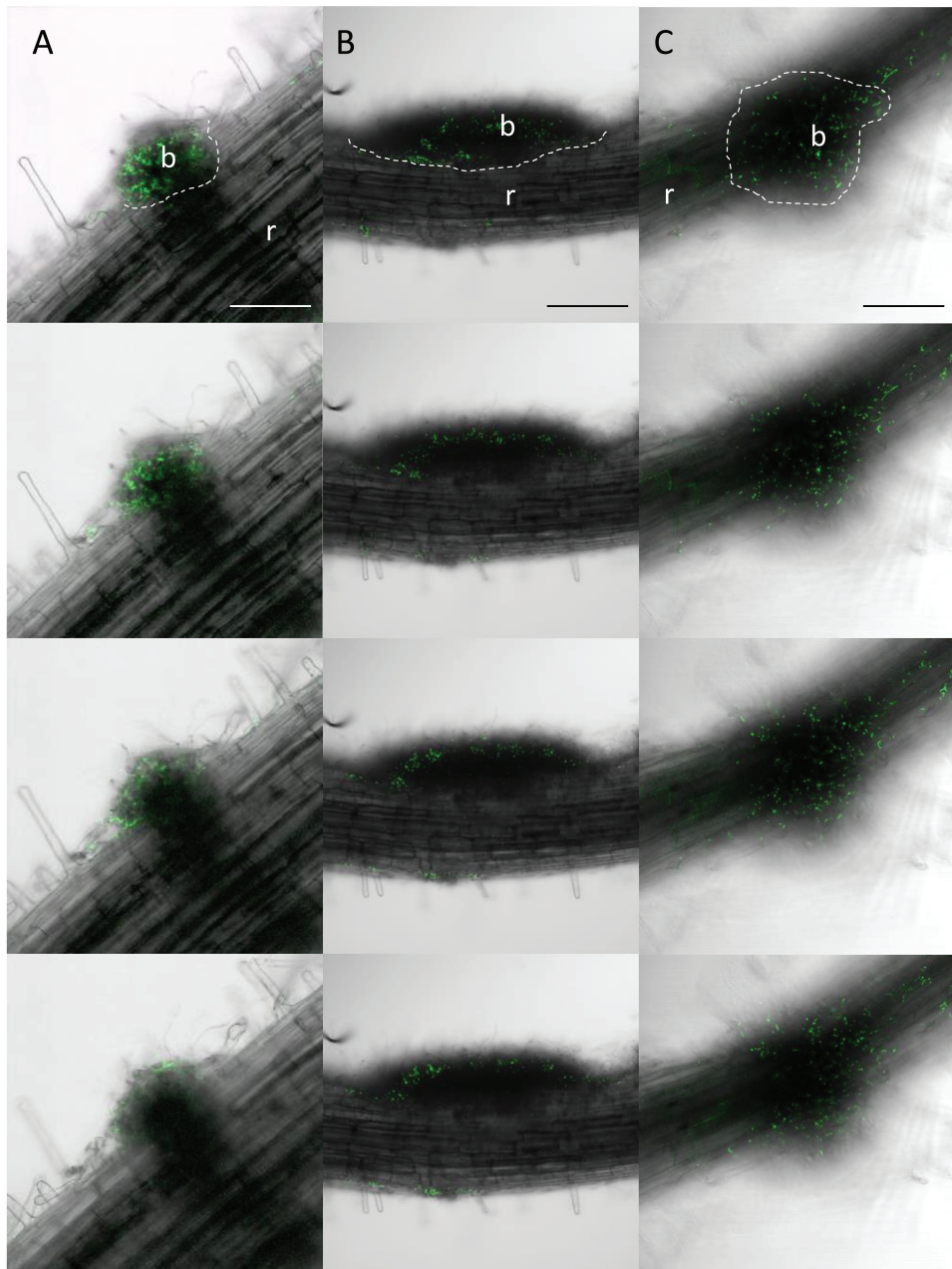

Supplement: Supplementary file 10 — Three representative optical section Z-series illustrating the preferential location of 1G6-GFP cells on different P. parasitica biofilms (b) formed on the surface of roots (r) (3 hpi). Fluorescence intensities were measured on ten consecutive serial sections (5 μm), (i) at the left and right parts of the biofilm (1000 μm2), and (ii) at the root surface not covered by P. parasitica and located on the left and right sides of the biofilm (1000 μm2). Bars: 100 μm in A and C; 200 μm in B. (PDF 29611 kb) [file 40168_2017_273_MOESM10_ESM.pdf]

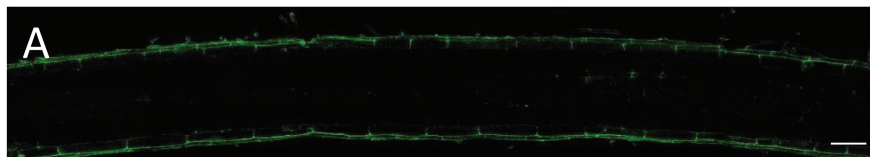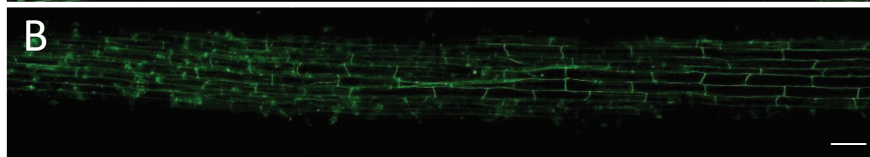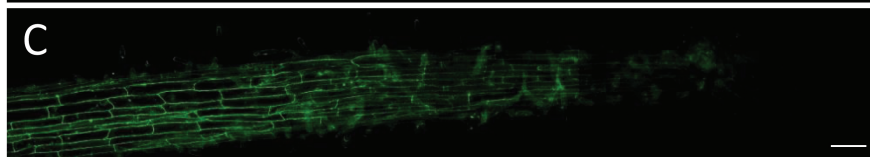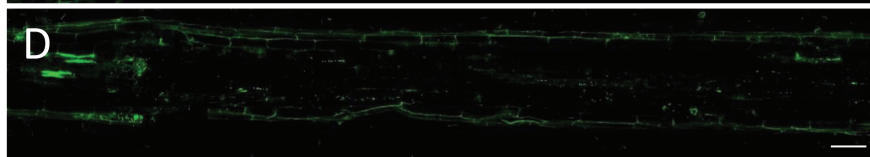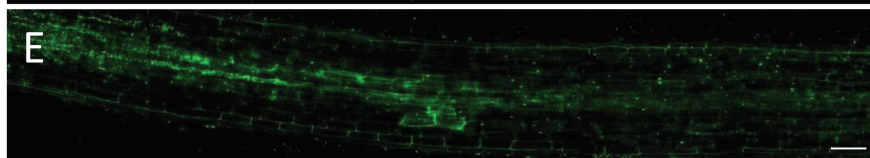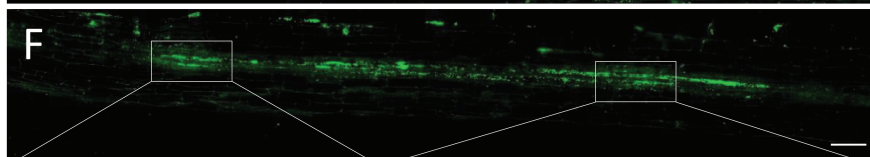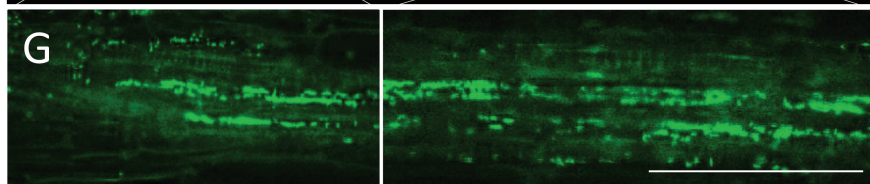

Supplement: Supplementary file 11 — Location of I-1G6-GFP in the root cortex at 8 dpi. (A, B, C) Roots nude (A) or recovered with P. parasitica biofilm (B, C) inoculated with E. coli-GFP cells. (D, E, F) Roots nude (D) or roots covered with P. parasitica biofilm (E, F) and inoculated with I-1G6-GFP. (G) Enlargement of the two inlets indicated in F, showing root cortex colonization along the longitudinal axis of the xylem. Bars: 100 μm. (PDF 24571 kb) [file 40168_2017_273_MOESM11_ESM.pdf]
