# Supplementary material for: Tomato root microbiota and Phytophthora parasitica-associated disease
Source: Microbiome. 2017 May 16;5:56. doi: 10.1186/s40168-017-0273-7 (PMC5434524; doi:10.1186/s40168-017-0273-7)
Supplement: Supplementary file 3 — Nucleotide sequences (5-3′) of the primers used in this study. (PDF 451 kb) [file 40168_2017_273_MOESM3_ESM.pdf]

Table S3

Rhizosphere bacterial families identified in M1 or M2, and responding to the presence of *P. parasitica* biofilm

		M1R1	M1R2	M1R3	M2R1	M2R2	M2R2	Mean M1	Mean M2	SD M1	SD M2	*p* value
**Proteobacteria**	**phylum**											
Alphaproteobacteria	class											
Rhizobiaceae	family	5,145	3,077	3,906	3,334	2,227	4,087	4,043	3,216	2,193	1,780	0,287
Hyphomicrobiaceae	family	1,229	1,487	1,304	0,634	0,949	0,954	1,340	0,846	0,679	0,449	0,022
Beijerinckiaceae	family	0,023	0,016	0,046	0,016	0,021	0,029	0,028	0,022	0,019	0,012	0,435
Rhizobiales_incertae_sedis	family	0,497	0,407	0,423	0,242	0,277	0,321	0,442	0,280	0,225	0,144	0,075
Bradyrhizobiaceae	family	0,427	0,524	0,704	0,262	0,341	0,397	0,552	0,333	0,299	0,176	0,039
Phyllobacteriaceae	family	0,159	0,208	0,207	0,123	0,102	0,167	0,191	0,131	0,098	0,071	0,115
Rhodobiaceae	family	0,033	0,017	0,043	0,018	0,016	0,026	0,031	0,020	0,019	0,011	0,171
Methylobacteriaceae	family	0,095	0,110	0,193	0,099	0,202	0,123	0,133	0,141	0,079	0,083	0,873
Brucellaceae	family	0,010	0,005	0,021	0,010	0,006	0,011	0,012	0,009	0,009	0,005	0,537
Methylocystaceae	family	0,024	0,030	0,055	0,016	0,037	0,025	0,037	0,026	0,023	0,016	0,423
Xanthobacteraceae	family	0,013	0,006	0,019	0,006	0,004	0,009	0,012	0,006	0,008	0,004	0,112
Aurantimonadaceae	family	0,006	0,002	0,025	0,009	0,002	0,017	0,011	0,010	0,011	0,008	0,786
Bartonellaceae	family	0,001	0,000	0,004	0,001	0,000	0,001	0,002	0,001	0,002	0,001	0,317
Caulobacteraceae	family	4,172	4,745	3,631	4,494	4,656	2,843	4,182	3,997	2,140	2,160	0,625
Sphingomonadaceae	family	8,167	7,145	5,787	3,668	4,229	3,738	7,033	3,878	3,649	1,955	0,048
Erythrobacteraceae	family	0,528	0,284	0,283	0,337	0,301	0,321	0,365	0,320	0,216	0,161	0,598
Rhodospirillaceae	family	0,623	0,734	1,029	0,378	0,814	0,714	0,795	0,635	0,433	0,368	0,318
Acetobacteraceae	family	0,081	0,053	0,066	0,039	0,061	0,062	0,067	0,054	0,035	0,029	0,490
Rickettsiaceae	family	0,268	0,284	0,437	0,166	0,071	0,152	0,330	0,130	0,182	0,077	0,064
Rhodobacterales	order	0,030	0,034	0,037	0,021	0,063	0,028	0,034	0,037	0,017	0,026	0,790
Rhodobacteraceae	family	0,030	0,034	0,037	0,021	0,063	0,028	0,034	0,037	0,017	0,026	0,790
Sneathiellaceae	family	0,024	0,010	0,058	0,021	0,026	0,034	0,031	0,027	0,025	0,014	0,766

		M1R1	M1R2	M1R3	M2R1	M2R2	M2R2	Mean M1	Mean M2	SD M1	SD M2	*p* value
Proteobacteria	***phylum***											
Betaproteobacteria	class											
Comamonadaceae	family	0,138	0,176	0,223	0,282	0,292	0,272	0,179	0,282	0,096	0,141	0,067
Burkholderiales_incertae_sedis	family	0,080	0,100	0,100	0,193	0,159	0,259	0,093	0,204	0,048	0,110	0,063
Oxalobacteraceae	family	0,024	0,050	0,064	0,049	0,036	0,174	0,046	0,086	0,028	0,076	0,389
Methylophilaceae	family	0,005	0,015	0,021	0,008	0,004	0,000	0,014	0,004	0,009	0,004	0,309

		M1R1	M1R2	M1R3	M2R1	M2R2	M2R2	Mean M1	Mean M2	SD M1	SD M2	*p* value
Proteobacteria	***phylum***											
Gammaproteobacteria	class											
Pseudomonadaceae	family	2,936	4,451	5,243	4,092	3,078	4,024	4,210	3,731	2,312	1,922	0,618
Xanthomonadaceae	family	0,010	0,013	0,015	0,008	0,006	0,011	0,013	0,008	0,007	0,005	0,090
Sinobacteraceae	family	2,358	3,252	5,098	2,770	7,459	3,281	3,569	4,503	2,118	3,079	0,648
Thiotrichaceae	family	0,187	0,336	0,563	0,210	0,235	0,229	0,362	0,225	0,238	0,113	0,318
Enterobacteriaceae	family	0,016	0,010	0,003	0,023	0,011	0,007	0,010	0,014	0,007	0,010	0,173
Chromatiaceae	family	0,045	0,189	0,054	0,093	0,144	0,037	0,096	0,091	0,081	0,063	0,874
Thiotrichales_incertae_sedis	family	0,040	0,179	0,038	0,088	0,140	0,029	0,086	0,086	0,079	0,062	0,999
Alteromonadaceae	family	0,067	0,057	0,147	0,015	0,021	0,017	0,090	0,018	0,061	0,009	0,130
Ectothiorhodospiraceae	family	0,068	0,104	0,054	0,062	0,093	0,019	0,075	0,058	0,043	0,042	0,198
Piscirickettsiaceae	family	0,068	0,104	0,054	0,062	0,093	0,019	0,075	0,058	0,043	0,042	0,198
Coxiellaceae	family	0,010	0,013	0,009	0,006	0,004	0,004	0,010	0,005	0,006	0,003	0,073

		M1R1	M1R2	M1R3	M2R1	M2R2	M2R2	Mean M1	Mean M2	SD M1	SD M2	*p* value
Proteobacteria	***phylum***											
Deltaproteobacteria	class											
Polyangiaceae	family	0,021	0,254	0,193	0,052	0,075	0,062	0,156	0,063	0,126	0,033	0,280
Phaselicystidaceae	family	0,007	0,028	0,154	0,005	0,010	0,009	0,063	0,008	0,072	0,005	0,347
Myxococcaceae	family	0,028	0,208	0,213	0,099	0,157	0,050	0,150	0,102	0,114	0,067	0,551
Cystobacteraceae	family	0,024	0,282	0,115	0,060	0,112	0,043	0,140	0,072	0,128	0,046	0,368
Kofleriaceae	family	0,004	0,053	0,046	0,004	0,018	0,008	0,034	0,010	0,028	0,008	0,181
Nannocystaceae	family	0,010	0,026	0,023	0,011	0,023	0,007	0,020	0,014	0,012	0,010	0,385
Bacteriovoracaceae	family	0,023	0,145	0,090	0,073	0,172	0,060	0,086	0,102	0,066	0,071	0,568
Bdellovibrionaceae	family	0,003	0,003	0,089	0,000	0,004	0,000	0,032	0,001	0,044	0,002	0,411

		M1R1	M1R2	M1R3	M2R1	M2R2	M2R2	Mean M1	Mean M2	SD M1	SD M2	*p* value
**Bacteroidetes**	**phylum**											
Flavobacteriaceae	family	41,240	26,005	19,071	44,755	34,522	46,201	28,772	41,826	9,213	6,367	0,079
Cryomorphaceae	family	0,728	0,656	0,504	0,367	1,022	0,358	0,630	0,582	0,114	0,381	0,423
Cytophagaceae	family	0,953	0,841	0,813	0,653	0,840	0,778	0,869	0,757	0,074	0,095	0,091
Flammeovirgaceae	family	0,051	0,067	0,057	0,045	0,125	0,032	0,058	0,067	0,008	0,051	0,387
Sphingobacteriaceae	family	0,507	0,716	1,022	0,282	0,500	0,266	0,748	0,350	0,259	0,131	0,038
Chitinophagaceae	family	0,200	0,570	0,307	0,209	0,341	0,176	0,359	0,242	0,190	0,087	0,193
Saprospiraceae	family	0,018	0,032	0,009	0,007	0,009	0,007	0,020	0,007	0,001	0,001	0,067
Cyclobacteriaceae	family	0,013	0,007	0,010	0,009	0,016	0,010	0,010	0,012	0,003	0,004	0,287
Prevotellaceae	family	0,009	0,009	0,002	0,003	0,009	0,007	0,007	0,006	0,004	0,003	0,440
