# Supplementary material for: Tomato root microbiota and Phytophthora parasitica-associated disease
Source: Microbiome. 2017 May 16;5:56. doi: 10.1186/s40168-017-0273-7 (PMC5434524; doi:10.1186/s40168-017-0273-7)
Supplement: Supplementary file 7 — 16S gene count statistics (20 March 2017). (PDF 445 kb) [file 40168_2017_273_MOESM7_ESM.pdf]

Table S5 16S gene count statistics (20 march 2017)

https://rrndb.umms.med.umich.edu/

Phylum	Genomes	Range	Mode	Median	Mean	Std Dev
Acidobacteria	18	[1 - 2]	1	1.0	1.3	0.5
Actinobacteria	687	[1 - 9]	4	3	3.3	1.7
Bacteroidetes	215	[1 - 13]	3	3	3.7	1.9
Chloroflexi	27	[1 -3]	1	1	1.5	0.7
Fibrobacteres	2	[3 - 3]	3	3	3.00	0.00
Firmicutes	1352	[1 - 16]	6	6	7	3.0
Fibrobacter	2	[3 - 3]	3	3	3.00	0
Gemmatimonadetes	3	[1 -2]	2	2	1.7	0.5
Nitrospirae	8	[1 - 3]	1	1.5	1.6	0.7
Planctomycetes	13	[1 - 8]	—	2	2.5	1.8
Proteobacteria	3424	[1 - 15]	7	4	4.7	2.5

Family	Genomes	Range	Mode	Median	Mean	Std Dev
Rhodospirillaceae	19	[1 - 10]	4	4	4.5	3.0
Sphingomonadaceae	44	[1 -3]		2	2.2	0.8
Caulobacteraceae	7	[1 - 3]	2	2	2.00	0.53
Hyphomicrobiaceae	14	[1 - 3]	1	1.5	1.7	0.8
Rhizobiaceae	116	[1-6]	3	3	2.6	1.0
Sinobacteriaceae	nd	nd	nd	nd	nd	nd
Pseudomonadaceae	229	[2 - 8]	4	4	4.8	1.2
Sphingobacteriaceae	10	[3 - 8]	4	4	5	1.7
Cryomorphaceae	1	[1 - 2]	2	2	2	0
Flavobacteriaceae	93	[1 - 13]	3	3	3.9	2.1
