# Supplementary material for: Tomato root microbiota and Phytophthora parasitica-associated disease
Source: Microbiome. 2017 May 16;5:56. doi: 10.1186/s40168-017-0273-7 (PMC5434524; doi:10.1186/s40168-017-0273-7)
Supplement: Supplementary file 8 — Phylogenetic trees showing the relationship between the Pseudomonadaceae (A) and Enterobacteriaceae (B) isolates from M2 and related Pseudomonadaceae or Enterobacteriaceae species, respectively. Trees were constructed using PhyML with default settings and 1000 bootstrap replicates. The position of rhizospheric isolates are indicated by vertical bars. (PDF 7876 kb) [file 40168_2017_273_MOESM8_ESM.pdf]

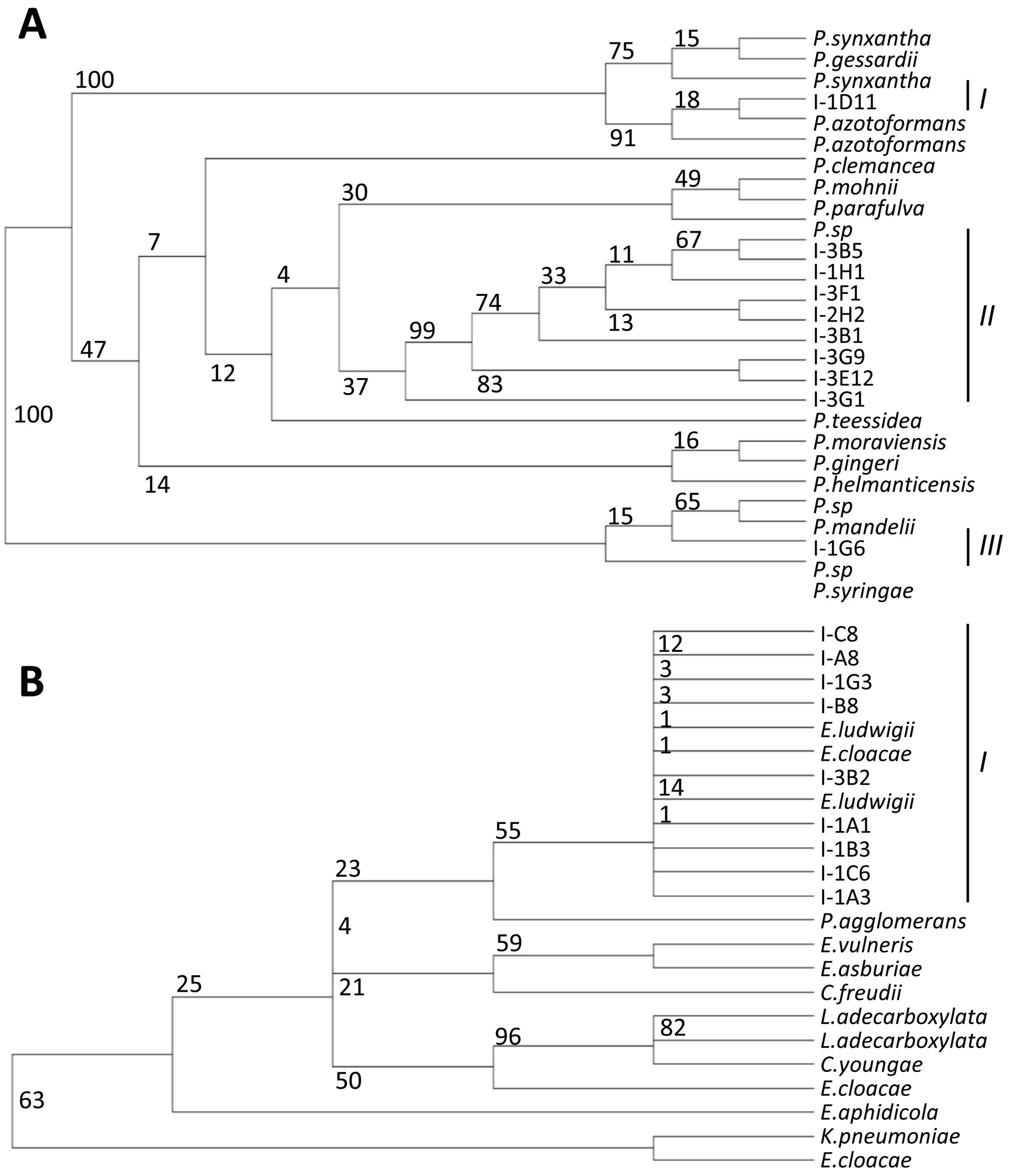
A
100
75
15
P.synxantha
P.gessardii
P.synxantha
I
18
I-1D11
P.azotoformans
91
P.azotoformans
P.clemancea
49
30
P.mohnii
P.parafulva
P.sp
67
I-3B5
I-1H1
11
33
I-3F1
7
4
74
II
I-2H2
99
13
I-3B1
47
I-3G9
12
37
83
I-3E12
I-3G1
100
P.teessidea
16
P.moraviensis
P.gingeri
14
P.helmanticensis
65
P.sp
15
P.mandelii
I-1G6
III
P.sp
P.syringae
B
I-C8
12
I-A8
3
I-1G3
3
I-B8
1
E.ludwigii
1
E.cloacae
I
I-3B2
14
E.ludwigii
1
I-1A1
55
I-1B3
23
I-1C6
I-1A3
4
P.agglomerans
59
E.vulneris
E.asburiae
25
21
C.freudii
L.adecarboxylata
82
96
L.adecarboxylata
C.youngae
50
E.cloacae
63
E.aphidicola
K.pneumoniae
E.cloacae
