# Supplementary material for: Tomato root microbiota and Phytophthora parasitica-associated disease
Source: Microbiome. 2017 May 16;5:56. doi: 10.1186/s40168-017-0273-7 (PMC5434524; doi:10.1186/s40168-017-0273-7)
Supplement: Supplementary file 9 — Two-week-old plants were first root-inoculated (A, C) or not inoculated (B, D) with a 10 ml suspension of zoospores (2 cells/μl) from the P. parasitica strain 149. Two replicates of five plants were subsequently inoculated with the indicated bacterial isolates and photographed 8 days post-inoculation (C, D). As shown in (C), when inoculated alone the tested isolates (I-3G9, I-1G6, I-1G3) did not cause visible disease symptoms on plants. As illustrated in (C), the co-inoculation of rhizospheric isolates with P. parasitica led to aggravation of symptoms when compared to the co-inoculation of E. coli cells with P. parasitica zoospores. (PDF 31934 kb) [file 40168_2017_273_MOESM9_ESM.pdf]

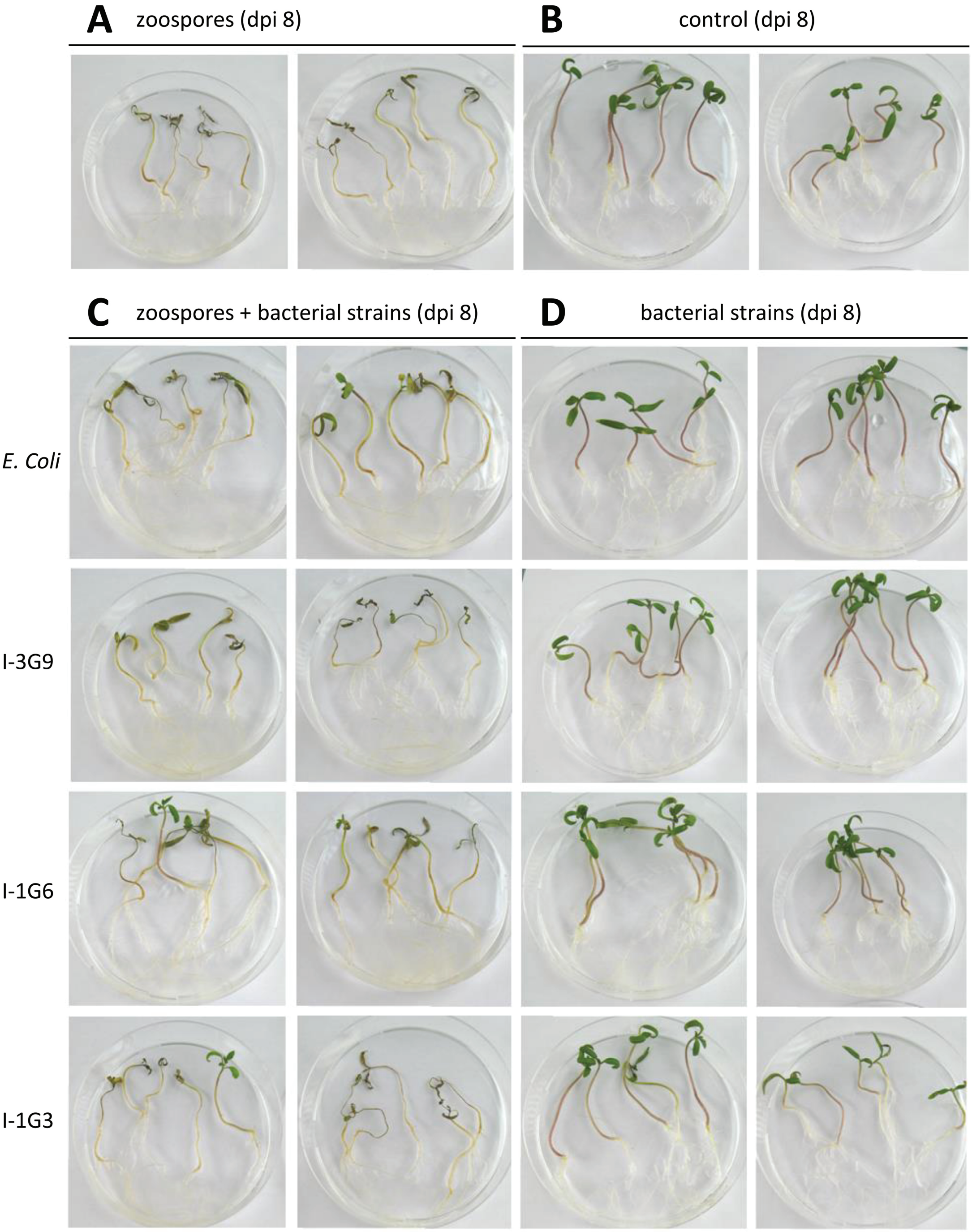

A
zoospores (dpi 8)
B
control (dpi 8)
C
zoospores + bacterial strains (dpi 8)
D
bacterial strains (dpi 8)
E. Coli
I-3G9
I-1G6
I-1G3
